# Agarwood Essential Oil Displays Sedative-Hypnotic Effects through the GABAergic System

**DOI:** 10.3390/molecules22122190

**Published:** 2017-12-09

**Authors:** Shuai Wang, Canhong Wang, Deqian Peng, Xinmin Liu, Chongming Wu, Peng Guo, Jianhe Wei

**Affiliations:** 1 Key Laboratory of Bioactive Substances and Resources Utilization of Chinese Herbal Medicine, Institute of Medicinal Plant Development, Chinese Academy of Medical Sciences & Peking Union Medical College, Beijing 100193, China; zhuizhirun@163.com; 2 Ministry of Education & National Engineering Laboratory for Breeding of Endangered Medicinal Materials, Institute of Medicinal Plant Development, Chinese Academy of Medical Sciences & Peking Union Medical College, Beijing 100193, China; 3 Hainan Provincial Key Laboratory of Resources Conservation and Development of Southern Medicine, Hainan Branch of the Institute of Medicinal Plant Development, Chinese Academy of Medical Sciences & Peking Union Medical College, Haikou 570311, China; xinzhuangjianpo@163.com; 4 Key Laboratory of State Administration of Traditional Chinese Medicine for Agarwood Sustainable Utilization, Hainan Branch of the Institute of Medicinal Plant Development, Chinese Academy of Medical Sciences & Peking Union Medical College, Haikou 570311, China; 5 School of Pharmacy, Hainan Medical College, Haikou 571199, China; pengdeqian2004@163.com; 6 Pharmacology and toxicology center, Institute of Medicinal Plant Development, Chinese Academy of Medical Sciences & Peking Union Medical College, Beijing 100193, China; liuxinmin@hotmail.com (X.L.); cmwu@implad.ac.cn (C.W.)

**Keywords:** agarwood essential oil, sedative-hypnotic effect, ethology, GABAergic system

## Abstract

Although agarwood has been used as a tranquilizer in Asian countries for hundreds of years, the underlying pharmacological basis is still unclear. This study investigated the sedative-hypnotic effect of agarwood essential oil (AEO) using locomotor activity and pentobarbital-induced sleeping assays in mice. Single (1-day) and multiple (7- and 14-days) administrations of 60 mg/kg AEO generated significant sedative effect on inhibiting locomotor activity and hypnotic effect on pentobarbital-induced sleeping in mice. Interestingly, prolonged AEO treatment did not result in obvious desensitization. Concoitant measurement of the levels of brain neurotransmitters using ultrafast liquid chromatography-tandem mass spectrometry (UFLC-MS/MS) indicated that AEO had no significant effect on the levels of glutamic acid (Glu) and γ-aminobutyric acid (GABA) in the brain. However, the sedative-hypnotic effects were blocked by the type A GABA (GABA_A_) receptor antagonists bicuculline and flumazenil. In addition, AEO significantly elevated the expression of GABA_A_ receptor subunits and subtypes in the cerebral cortex. Furthermore, AEO increased chlorine ion (Cl^−^) influx through GABA_A_ receptors in human neuroblastoma cells. These results together demonstrate that AEO exerts its sedative-hypnotic effects through regulating gene expression of GABA_A_ receptors and potentiating GABA_A_ receptor function.

## 1. Introduction

Agarwood, a highly precious fragrant non-timber forest product, has been used for centuries in fragrances, incense, medicines, aromatherapy, and religion [1,2,3,4]. Due to its unique aroma, agarwood is used for incense and aromatherapy in the Middle East, and burning for fragrance in Japan. Agarwood has also been widely used as a medicine for tranquilizing and reducing excitement in East and South Asia for nearly two centuries. For example, the Compendium of Materia Medica, a pharmaceutical masterpiece written by Shizhen Li during the Ming Dynasty, recorded agarwood being used for treating forgetfulness and fright [5]. However, to our knowledge, only four studies have reported the sedative-hypnotic function of agarwood. Okugawa et al. [6] reported that agarwood benzene extract reduced spontaneous motility and prolonged hexobarbital-induced sleeping time. Further phytochemical and pharmacological screening showed that jinkoh-eremol and agarospirol had a neuroleptic effect [7]. Agarwood essential oil (AEO), generally considered to have anticancer, anti-inflammation, and anti-oxidant activities [8,9,10], also demonstrated a sedative effect on mice through inhalation. Takemoto et al. [11] reported that AEO could sedate mice by vapor inhalation. Miyoshi et al. [12] revealed that benzylacetone, released by heated agarwood, had sedative activity in mice. However, obtaining an exact dose-effect relationship is difficult with drug administration via inhalation. Furthermore, the mechanism of agarwood’s sedative-hypnotic function has not yet been reported. The few investigations on the sedative-hypnotic function of agarwood are largely dissymmetric with the various applications in large quantities. One of the main reasons for the lack of studies might be that the material is becoming hard to obtain from wild sources, and the price is too high.

Sleep disorders, like insomnia, are a growing mental health problem, affecting the quality of life and frequently causing significant functional impairment [13]. Benzodiazepines and benzodiazepine receptor agonists are the most widely used clinical controller of sleep problems, which were introduced into clinical practice in the 1960s [14,15]. Benzodiazepines and benzodiazepine receptor agonists act by binding to the benzodiazepine-receptor binding site of the type A γ-aminobutyric acid (GABA_A_) receptor, increasing the activity of the inhibitory neurotransmitter GABA, and enhancing inhibitor outputs to all major cell groups in the brainstem and the hypothalamus that promotes arousal. However, long-term exposure to benzodiazepine usually results in many undesirable side-effects, such as dependence and tolerance [15,16,17]. Therefore, substances that could contribute to inducing and improving sleep quality with fewer side-effects would be beneficial.

With the use of artificial methods, such as the whole-tree agarwood-inducing technique for producing agarwood [18], the agarwood yields are increasing and the price is becoming more reasonable. As a result, agarwood no longer needs to be obtained from wild natural resources, enabling its wider application. To further investigate the sedative-hypnotic function of agarwood, we performed systematic investigations. The sedative-hypnotic effect of AEO was evaluated through animal behavioral tests, and the potential mechanism on GABAergic system was explored.

## 2. Results

### 2.1. AEO Component Analysis

**Figure 1:**
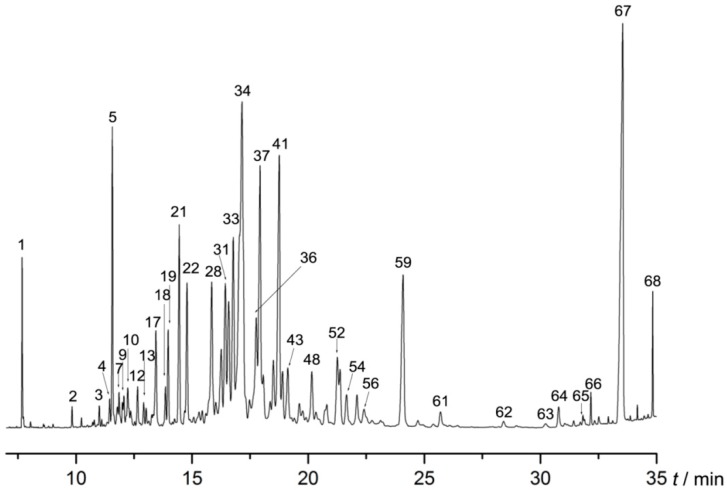
Gas chromatograph mass spectrometry (GC-MS) chromatograms of agarwood essential oil (AEO).

Sixty-eight compounds (Table 1 and Figure 1), representing 98.244% of the AEO, were identified by comparing their MS data with database data. Thirty-four sesquiterpenes, which included gualol (14.089%), dehydrofukinone (4.096%), aristolene (4.063%), 6-isopropyl-4,8-α- dimethyl-1,2,3,7,8,8-hexahydronaphthalene (3.481%), 2,3,3,3,4,5-hexahydro-7-isopropyl-3-methyl-1*H*-cyclopenta[1,3]cyclopropa-[1,2]benzene-3,6(7*H*)-dione (3.175%), germacrene B (3.121%), sandal (2.996%), 1,1,7-trimethyl-4-methylenedecahydro-1*H*-cyclopropa[e]azulene (2.874%) and hinesol (2.745%). were the main (51.132%) components of AEO. Thirteen compounds were aromatics (24.114%), including 7-methyltridecane (11.228%), 2,3,4,5-tetramethyltricyclo[3.2.1.02,7]oct-3-ene (5.716%), pyrethrone (2.316%) and perhydropyrene (1.619%). Other known compounds in the essential oil accounted for 19.823%.

### 2.2. Effect of AEO on Locomotor Activity in Mice

**Figure 2:**
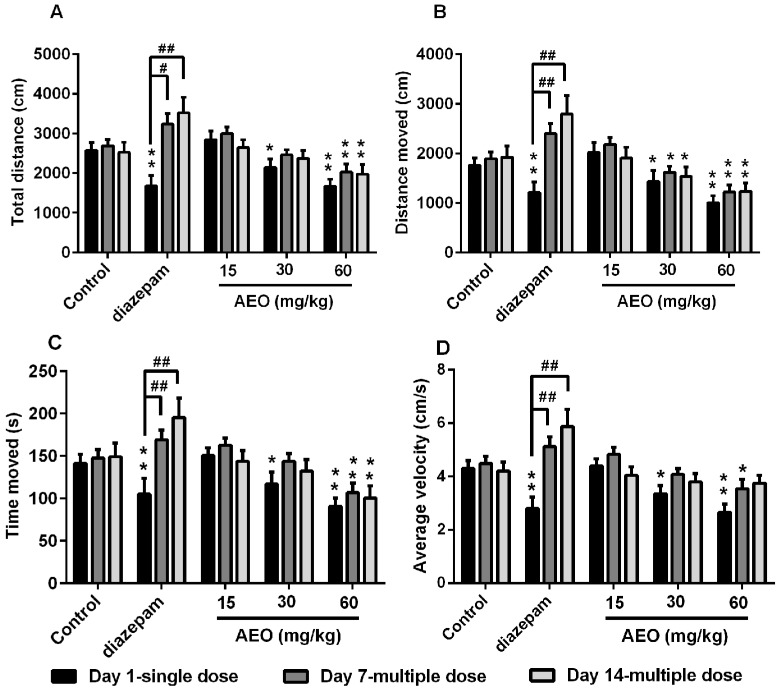
Effect of AEO on locomotor activity of mice: (**A**) total distance, (**B**) distance moved, (**C**) time moved and (**D**) average velocity were recorded over a 10-min-session, 30 min after intraperitoneal (i.p.) administration of AEO (15, 30, and 60 mg/kg), diazepam (5 mg/kg), or saline. The results are presented as mean ± SEM with *n* = 12, * *p* < 0.05, and ** *p* < 0.01 vs. control group at the same time. # *p* < 0.05 and ## *p* < 0.01 vs. corresponding group at the same dose.

Treatment with AEO significantly decreased total distance traveled in a dose-dependent manner after single administration (Figure 2A). A single administration of AEO significantly and dose-dependently reduced distance moved, time moved and average velocity of mice (Figure 2B–D). The positive control diazepam, a commonly used clinical sedative drug, also displayed a sedative effect on total distance, distance moved, time moved and average velocity, which all significantly decreased. However, over time, the sedative effect of diazepam decreased after multiple (7 days and 14 days) injections, which is consistent with previous reports [15,19]. Interestingly, after multiple administrations of AEO, even after 7 or 14 days, sedative effects were maintained without obvious desensitization compared to a single dose.

### 2.3. Effect of AEO on Pentobarbital-Induced Sleeping in Mice

**Figure 3:**
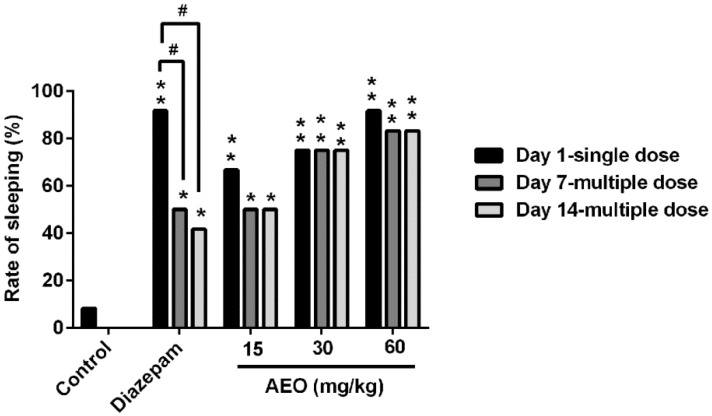
Effect of AEO on subthreshold pentobarbital-induced sleeping. AEO (15, 30, and 60 mg/kg), diazepam (2 mg/kg), or saline was administrated i.p. 30 min before the pentobarbital sodium (25 mg/kg) i.p. injection. The results are presented as mean ± SEM with *n* = 12. * *p* < 0.05 and ** *p* < 0.01 vs. control group at the same time. # *p* < 0.05 vs. the corresponding group at the same dose.

Compared to the control group (Figure 3), a single administration of AEO (15, 30, and 60 mg/kg) and diazepam (2 mg/kg) significantly increased the rate of sleeping induced by the subthreshold pentobarbital sodium. The effect of AEO showed dose-dependent enhancement. Additionally, the hypnotic function in mice was largely maintained after multiple administrations of AEO, both on day 7 and day 14, without significant differences compared to a single dose, whereas the function of diazepam (2 mg/kg) obviously decreased (Figure 3).

**Figure 4:**
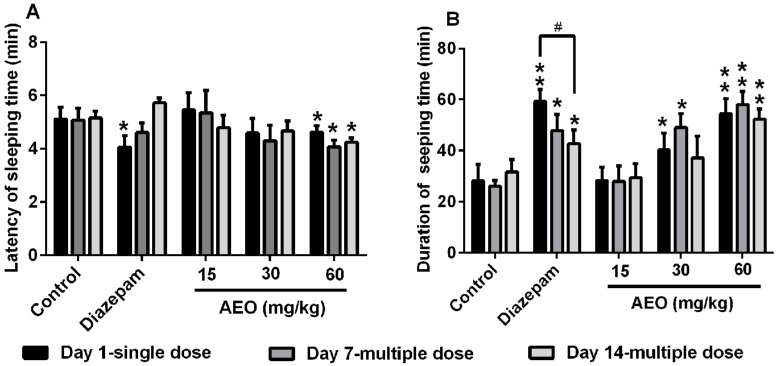
Effect of AEO on the hypnotic dose of pentobarbital-induced sleeping: (**A**) latency of sleeping time and (**B**) duration of sleeping time were recorded. AEO (15, 30, and 60 mg/kg), diazepam (2 mg/kg), or saline was administrated i.p. 30 min before the pentobarbital sodium (50 mg/kg) i.p. injection. The results are presented as mean ± SEM with *n* = 12. * *p* < 0.05 and ** *p* < 0.01 vs. control group at the same time. # *p* < 0.05 vs. the corresponding group at the same dose.

For the hypnotic dose of the pentobarbital-induced sleeping assay, as shown in Figure 4, the results revealed that AEO decreased the latency of sleeping time and prolong the duration of sleeping time in mice in a dose-dependent manner. Treatment with AEO (60 mg/kg) significantly reduced the latency of sleeping time, regardless of length of treatment (1, 7, or 14 days), whereas diazepam (2 mg/kg) only had that effect with single administration (Figure 4A). Similarly, AEO dose-dependently increased the duration of sleeping time with single and multiple administrations without obvious desensitization, whereas the potency of diazepam (2 mg/kg) decreased as administration time increased and significant tolerance appeared between day 1 and day 14 (Figure 4B).

### 2.4. Effect of AEO on the Brain Neurotransmitters Levels

**Figure 5:**
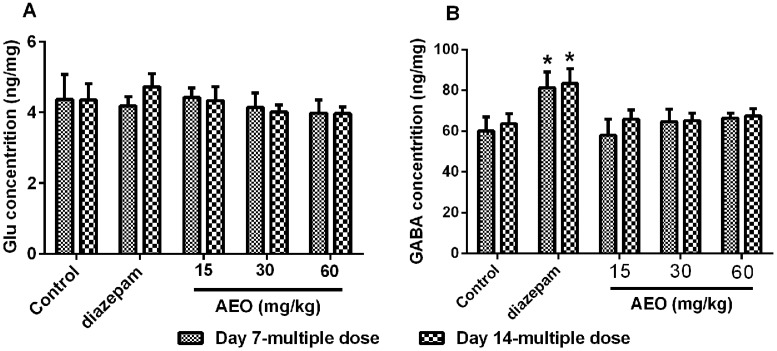
Effect of AEO on brain neurotransmitters in mice. Concentration of (**A**) glutamic acid (Glu) and (**B**) γ-aminobutyric acid.(GABA) were analyzed by ultrafast liquid chromatography-tandem mass spectrometry (UFLC-MS/MS). The cerebral cortex tissues were collected after a locomotor activity test. The results are presented as mean ± SEM with *n* = 6. * *p* < 0.05 vs. control group at the same time.

Neurotransmitters play a critical role in intercellular neural signal transmission, and many sedative-hypnotic agents usually exert their pharmacological effects by altering the concentration of neurotransmitters. So, we assessed the neurotransmitters in the cerebral cortex of the mice by using an ultrafast liquid chromatography-tandem mass spectrometry (UFLC-MS/MS) system. The results, as shown in Figure 5, demonstrated that AEO did not have obvious effects on the concentration of glutamic acid (Glu) and GABA. Diazepam significantly elevated the concentration of GABA and did not have an apparent influence on the Glu level (Figure 5).

### 2.5. Effects of GABA_A_ Receptor Antagonist on Locomotor Activity and Pentobarbital-Induced Sleeping

**Figure 6:**
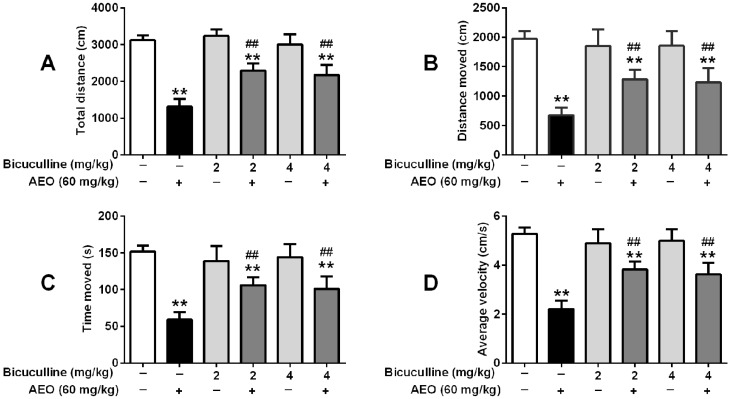

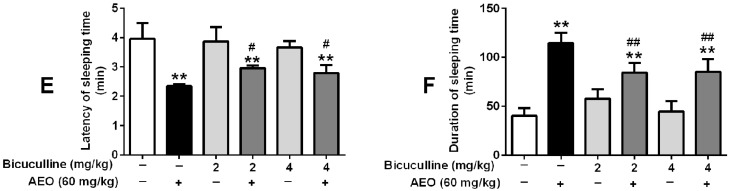
Effects of the GABA_A_ receptor antagonist bicuculline on locomotor activity and pentobarbital-induced sleeping: (**A**) total distance (**B**) distance moved, (**C**) time moved and (**D**) average velocity were recorded over a 10-min session, 30 min after i.p. administration of AEO (60 mg/kg) or saline, followed by (**E**) latency of sleeping time and (**F**) duration of sleeping time recorded after the pentobarbital sodium (50 mg/kg) i.p. injection. The results are presented as mean ± SEM with *n* = 8. ** *p* < 0.01 vs. control. # *p* < 0.05 and ## *p* < 0.01 vs. AEO 60 mg/kg group.

**Figure 7:**
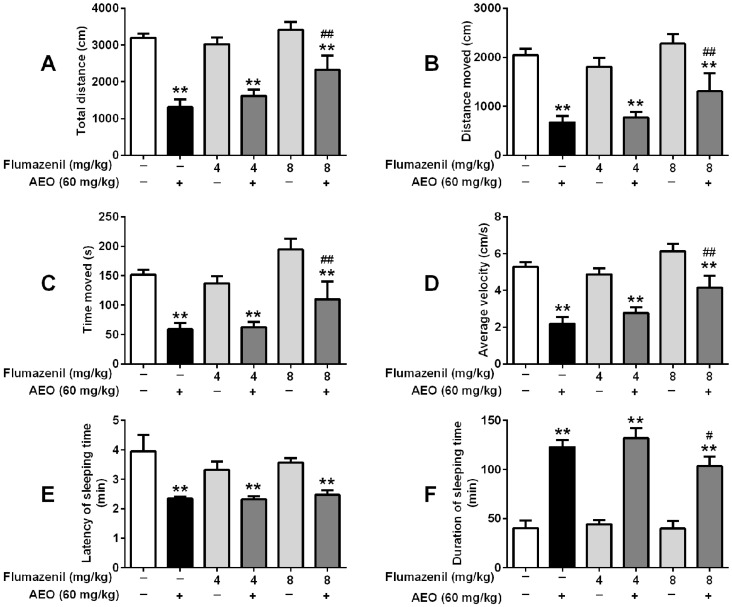
Effects of the GABA_A_ receptor antagonist flumazenil on locomotor activity and pentobarbital-induced sleeping: (**A**) total distance (**B**) distance moved, (**C**) time moved and (**D**) average velocity were recorded over a 10-min session, 30 min after i.p. administration of AEO (60 mg/kg), followed by (**E**) latency of sleeping time and (**F**) duration of sleeping time recorded after the pentobarbital sodium (50 mg/kg) i.p. injection. The results are presented as mean ± SEM with *n* = 8. ** *p* < 0.01 vs. control. # *p* < 0.05 and ## *p* < 0.01 vs. AEO 60 mg/kg group.

Even though AEO did not have obvious effect on the concentration of GABA, we supposed that AEO might act on the GABA receptor. So we used bicuculline and flumazenil to investigate the influence of AEO on locomotor activity and pentobarbital-induced sleeping. As the results in Figure 6 and Figure 7 show, both bicuculline (2 mg/kg and 4 mg/kg) and flumazenil (4 mg/kg and 8 mg/kg) antagonized the action of AEO. Only treatment with bicuculline had an indistinct influence on locomotor activity and pentobarbital-induced sleeping. Interestingly, when AEO (60 mg/kg) was administrated with bicuculline, the mice locomotor activities, including total distance, distance moved, time moved, and average velocity significantly increased, pentobarbital-induced latency of sleeping time increased, and duration of sleeping time decreased compared to the administration of AEO only (Figure 6).

Similarly, with a single injection of flumazenil, no apparent influence was observed on mice locomotor activity or sleeping induced by pentobarbital sodium. When treated with AEO (60 mg/kg) along with flumazenil, the sedative and sleep-promoting effect of AEO decreased along with the total distance, distance moved, time moved and average velocity increased, and duration of sleeping time decreased (Figure 7).

### 2.6. Effects of AEO on mRNA Expression of GABA_A_ Receptors Subunits and Subtypes

**Figure 8:**
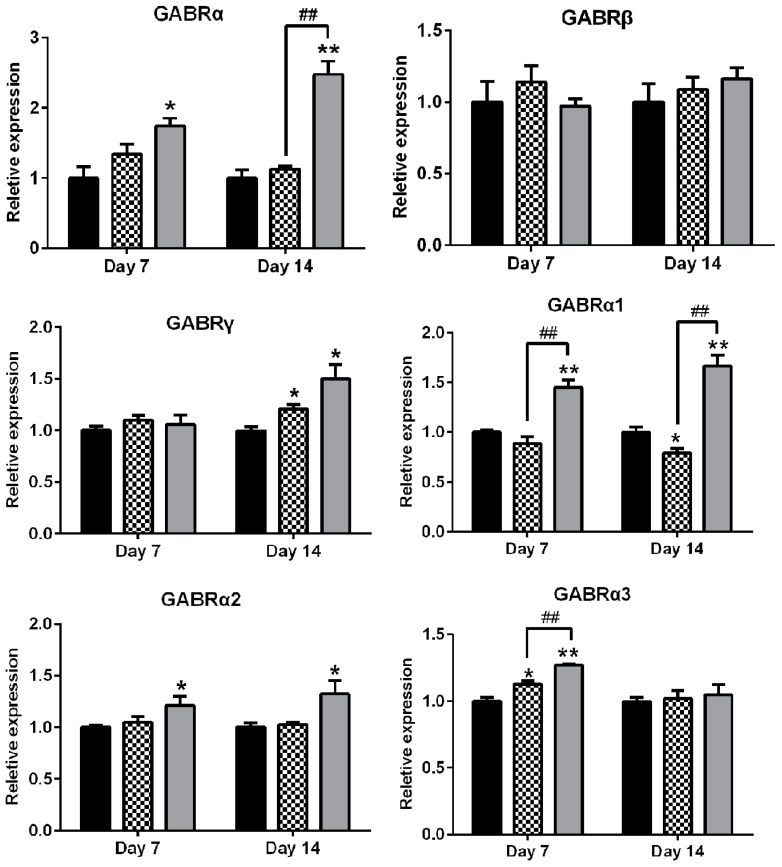

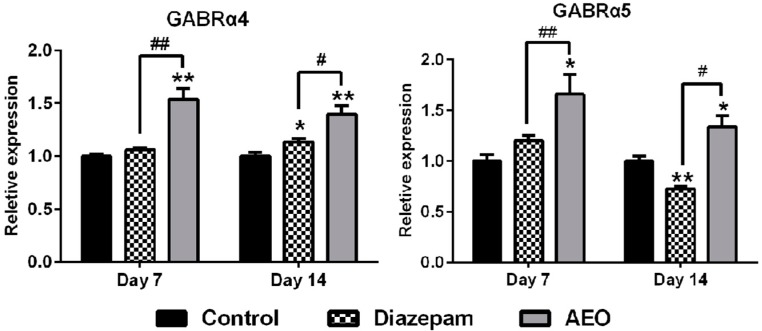
Effects of AEO (60 mg/kg) and diazepam (5 mg/kg) on mRNA expression of GABA_A_ receptor subunits and subtypes in mice. Values are represented as mean ± SEM. Results are representative of three independent experiments with *n* = 3. * *p* < 0.05 and ** *p* < 0.01 vs. control. # *p* < 0.05 and ## *p* < 0.01 vs. diazepam group at the corresponding time.

Based on the above results, quantitative real time polymerase chain reaction (RT-PCR) was used to investigate the mRNA level of GABA_A_ receptor subunits and subtypes in the cerebral cortex of the mice in two rounds. In the first round, we tested the expression of GABA_A_ receptor subunits α, β, and γ with subunit-nonspecific primers. As shown in Figure 8, AEO significantly increased the mRNA expression of the α subunits on both day 7 and day 14, whereas diazepam had no obvious effect on subunit α. Additionally, subunits γ were increased by AEO and diazepam on day 14, but not on day 7. No difference between AEO and diazepam on subunit β regulation was found compared with the control on day 7 or 14. Based on the results, subunit-specific primers were used in the second round to determine the difference in the subtypes of α-subunits, including α1 α2, α3, α4, and α5. The results showed that AEO considerably increased α subtype 1 to 5 both on day 7 and 14, except for α3 on day 14. Conversely, diazepam mostly increased α3 subunit on day 7, α4 subtype on day 14, and decreased α1 and α5 on day 14.

### 2.7. Effects of AEO on Cl^−^ Influx in SH-SY5Y Cells

**Figure 9:**
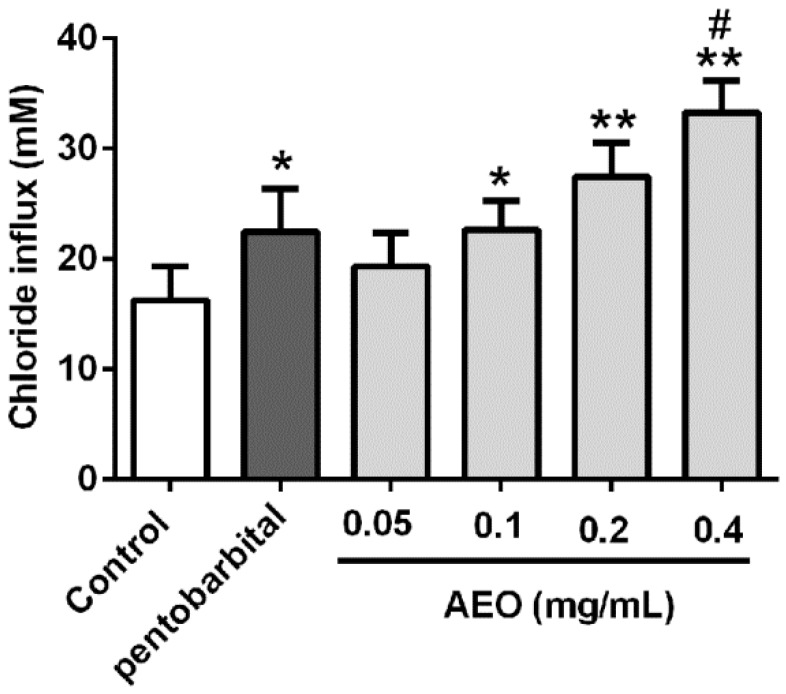
Effects of AEO on chlorine ion (Cl^−^) influx in SH-SY5Y cells. The results are presented as mean ± SEM with *n* = 8. * *p* < 0.05 and ** *p* < 0.01 vs. control group. # *p* < 0.05 vs. pentobarbital group.

To demonstrate the effect of AEO on GABA_A_ receptor function, intracellular Cl^−^ concentration was tested using a Cl^−^ fluorescence probe *N*-(Ethoxycarbonylmethyl)-6-methoxyquinolinium bromide (MQAE) in SH-SY5Y cell. As shown in Figure 9, both AEO and pentobarbital exhibited significant promoting activities on the Cl^−^ influx compared to the control. Treatment with AEO (0.05, 0.1, 0.2, and 0.4 mg/mL) dose-dependently increased the Cl^−^ influx, and AEO (0.1 and 0.2 mg/mL) had comparable potentiation with pentobarbital (0.1 mg/mL), whereas higher concentrations of AEO (0.4 mg/mL) were more efficient than pentobarbital (0.1 mg/mL) on Cl^−^ influx.

## 3. Discussion

**Figure 10:**
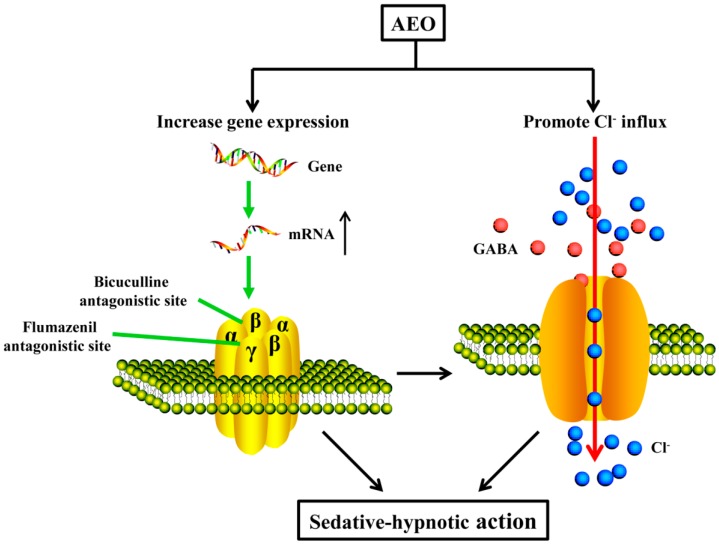
A schematic illustration of the proposed AEO mechanism.

This study demonstrated that AEO has a sedative-hypnotic effect through multiple animal behavior tests over different time periods range, which may contribute to the understanding of scientific nature of the traditional agarwood application. Furthermore, this study revealed that the mechanism of AEO on sedative-hypnotic function may potentially be related to the GABAergic system regulation (Figure 10).

### 3.1. AEO Had Sedative-Hypnotic Effect in Mice

AEO has been reported to inhibit spontaneous motor activity in mice after vapor inhalation [11]. Our previous results also demonstrated that AEO had a sedative-hypnotic effect on mice through inhalation [20]. The advantage of inhalation is avoiding the first pass hepatic metabolism and the drug rapidly transforms into the blood for faster efficacy. However, the disadvantage is that the drug administration dosage is hard to control, leading to an undefined dosage-effect relationship. In this study, we used intraperitoneal injection which avoids the first pass hepatic metabolismsimilar to inhalation, and takes advantage of the most common application of agarwood as incense.

As per the previous reports, the sedative-hypnotic effect of agarwood was assessed after a single dose administration of agarwood extracts or AEO [6,11]. We evaluated the function of AEO on locomotor activity and pentobarbital-induced sleeping in mice with a single dose intraperitoneal injection. The results indicated that AEO could significantly reduce locomotor activity in a dose-dependent manner (Figure 2), in accordance with inhalation results in previous reports [11,20]. Generally, a decrease in the locomotor activity of mice is indicative of a sedative action of pharmacological drugs [13] and this behavioral alternation is considered as a reflection of decreased excitability in the central nervous system [21]. The inhibitive effect on locomotor activity showed that AEO has sedative and excitability suppressive functions. Simultaneously, AEO demonstrated a synergic effect with pentobarbital, with an increased rate of sleeping (Figure 3), an increased duration of sleeping time and a decreased latency of sleeping time (Figure 4), which revealed the hypnotic-like effect of AEO. Prolonged administration of sedative-hypnotic drugs, such as diazepam, usually creates tolerance. We assessed the effect of AEO by multiple dose applications after 7 and 14 days. Interestingly, the results showed that AEO sustained the sedative-hypnotic action after multiple treatments without apparently increasing locomotor activity (Figure 2), decreasing the rate of sleeping (Figure 3), or significantly reducing the duration of sleeping time (Figure 4B). Conversely, the effect of diazepam decreased (Figure 3 and Figure 4) or even disappeared completely (Figure 2) after prolonged injection, is accordance with a previous report [19]. Overall, the results verified the sedative-hypnotic function of AEO in a dose-dependent manner without apparent tolerance development after prolonged administration.

### 3.2. Sedative-Hypnotic Effect Related to GABAergic System Regulation

GABAergic system is the target of several pharmacologically relevant drugs, like benzodiazepines on sedative-hypnotic functioning [14,15]. We used bicuculline, a competitive antagonist of the GABA_A_ receptor, and flumazenil, a specific antagonist of the benzodiazepine site in the GABA_A_ receptor complex, to investigate the influence of the GABA_A_ receptor on the AEO sedative-hypnotic function. The result showed that bicuculline inhibited the sedative-hypnotic effect of AEO (Figure 6), revealing that AEO acts through the GABA_A_ receptors in that it shows GABA-like function. Simultaneously, flumazenil dose-dependently blocked the effect of AEO (Figure 7), demonstrating that the active compounds in AEO influences the benzodiazepine site. Overall, these results indicated that AEO could act through the GABA_A_ receptor.

To further investigate the effect of AEO on the GABA_A_ receptor, the expression of GABA_A_ receptors subunits in mice cortexes were detected by RT-PCR. The results showed that AEO significantly increased the GABA_A_ receptor α subunits and α-subtypes including α1, α2, α3, α4, and α5 on day 7, and elevated α subunits, β subunits, and α-subtype α1, α2, α4, and α5 expression on day 14, whereas diazepam only promoted the expression of β subunits and α4, and even inhibited α-subtype α1 and α5 on day 14 (Figure 8). Therefore, the results demonstrate that AEO might work by increasing the expression of GABA_A_ receptor-associated genes to contribute to the function of GABA_A_ receptors. Simultaneously, AEO appeared to have a different regulative effect from diazepam. Tolerance to the sedative effect of benzodiazepine has been reported to be associated with the expression of aberrant GABA_A_ receptors [15,22], and numerous studies have reported changes in the expression of GABA_A_ receptor subunits [23,24,25]. The different regulative function of AEO from diazepam on GABA_A_ receptor gene expression might be the reason for the lack of tolerance development, even though the number of GABA_A_ receptor need to be further studied.

The GABA_A_ receptor functions through Cl^−^ influx, so the AEO promotion of the Cl^−^ influx was measured in human neuroblastoma cells. As shown in Figure 9, AEO significantly increased the Cl^−^ influx in a dose-dependent manner. These data demonstrate that the sedative-hypnotic effects of AEO are mediated through promoting the function of GABA_A_ receptors. Both the acting on GABA_A_ receptors and promoting the receptor gene transcription enhance the function of the GABA_A_ receptors, which was participated by promoting the Cl^−^ influx (Figure 10). However, AEO did not have an apparent action on Glu and GABA concentration in cerebral cortex (Figure 5). The reason for this might be that AEO does not influence the synthesis and metabolism of the neurotransmitters.

Based on the above result, we analyzed the components of the AEO using gas chromatograph mass spectrometry (GC-MS). As shown in Figure 1 and Table 1, a total of 68 compounds were identified, accounting for 98.244% of the AEO, in which many active compounds with neural regulative function were found, including benzylacetone (compound **1** in Table 1), which has been reported to have a sedative effect on mice [12], butylated hydroxytoluene [26] (compound **12**) and farnesene [27] (compound **24**) which are known to have a neuroprotective effect. Additionally, dehydrofukinone (compound **59**) has been proven to be a sedative or anesthetic agent through the GABA_A_ receptor in fish, and has suppressed neuronal excitability through GABAergic neuronal inhibition in mice [28,29]. Verbenol (compound **10**) has shown action on δ subunit-containing GABA_A_ receptors [30]. To some extent, this evidence has demonstrated that active compounds participate in the regulation of the GABAergic system, which may contribute to the sedative-hypnotic function of AEO.

### 3.3. Possible Applications and Limitations

This study highlighted the sedative-hypnotic function of AEO, which might have promising application for the prevention and treatment of sleep disorders. The results also scientifically interpreted the traditional application of AEO for tranquilizing and reducing excitement, which might promote the use of incense and new drug development from AEO and agarwood. However, this investigation applied AEO, rather than a single compound, through intraperitoneal injection, rather than inhalation, which might be different compared to daily application. In the following study, we will focus on a single compound during dose-controlled inhalation. Additionally, AEO might act on other targets or pathways other than the GABAergic system, which will require further exploration in the future.

## 4. Materials and Methods

### 4.1. AEO Preparation and Chemical Analysis

The agarwood, produced with the whole tree agarwood–inducing technique [18], was purchased from Maoming (Guangdong, China) and was identified by Prof. Jian-he Wei. It was smashed and soaked in water overnight. The AEO was extracted by hydrodistillation for 12 h, dried over anhydrous sodium sulfate (Na_2_SO_4_) and stored in a freezer at −20 °C until analysis. The AEO was diluted with *n*-hexane (high performance liquid chromatography (HPLC) grade) and injected into a gas chromatograph (GC) equipped with a HP-5MS capillary column (5% phenylmethylsiloxane, 30 m × 0.25 mm i.d., film thickness 0.25 μm) and a mass spectrometer with an ion trap detector (Agilent Technologies, Santa Clara, CA, USA) in full scan mode under electron impact ionization (70 eV). The gas carrier was helium at a flow rate of 1 mL/min. The injections were performed in splitless mode at 240 °C. The operating parameters were the temperature program of 50 °C for 1 min, an increase from 10 °C to 140 °C, maintained at 140 °C for 2 min, and a subsequent increase to 350 °C. The scan range was 40–500 amu under full scan. Most constituents were analyzed by GC-MS. Identification was completed by comparing their mass spectra with these stored in NIST 11 database and MSD Chemstation. The relative contents of the chemical components were calculated using the peak area normalization method.

### 4.2. Animals

Adult male Institute of Cancer Research (ICR) mice, weighting 18–20 g, were provided by the Vital River Laboratories (Beijing, China, License No. SYXK 2013-0023). All animals were housed in plastic cages in a controlled environment with a temperature of 23 ± 2 °C and a 60 ± 5% humidity with an alternating 12 h light, 12 dark cycle, with access to water and diet *at libitum*. All behavioral evaluations were performed during the day (8:00 a.m. – 8:00 p.m.). The animal experiments were performed in accordance with the NIH Guide for the Care and Use of Laboratory Animals and performed under the approval and supervision of the Animal Ethics Committee of the Institute of Medicinal Plant Development, Chinese Academy of Medical Sciences.

### 4.3. Reagents

Pentobarbital sodium, dimethyl sulfoxide (DMSO), and all calibration standards employed in the brain neurotransmitters determination were bought from Sigma (St. Louis, MO, USA). Bicuculline, flumazenil and diazepam were purchase from the National Institute for Food and Drug Control (Beijing, China). All chemicals and reagents used in the cell culture were from Gibco (Gaithersburg, MD, USA). Pentobarbital sodium was dissolved in saline. Diazepam, bicuculline and flumazenil were prepared in saline consisting of 1% Tween 80. AEO was first dissolved in DMSO at the concentration of 0.5 g/ml, and then suspended in saline containing 1% Tween 80 based on a dose of 15, 30 and 60 mg/kg. All drugs were administrated intraperitoneally at 10 mL/kg.

### 4.4. Locomotor Activity Test

A computer-aided controlling system that consisted of four cages (35 × 35 × 30 cm, length × width × height) with a 120 Lux light source and a video camera at the top was used to assess the mice locomotor activity as previously reported [19,31]. AEO (15, 30 and 60 mg/kg) and diazepam (5 mg/kg) were administrated i.p. 30 min before the experiment. The animals were placed in the cages individually for 2 min to acclimatize. The movement of mice was recorded for 10 min using cameras with a computer-aided image-processing system. The appropriate movement threshold was selected as 6.5 cm/s, and the total distance, distance moved, time moved and average velocity were collected to reveal the sedative action of AEO.

### 4.5. Pentobarbital-Induced Sleeping

A subthreshold dose of pentobarbital treatment was used to test the rate of sleeping, and a hypnotic dose was applied to gain the latency and duration of sleeping time, as previous described with little modification [32]. Pentobarbital sodium solution was injected i.p. at the sub-hypnotic (25 mg/kg) or hypnotic (50 mg/kg) dose after administrated (i.p.) of saline or drugs. The disappearance of righting reflex beyond 30 s was considered as asleep. The rate of sleeping was calculated as follows: rate of sleeping (%) = asleep number/total number × 100%. Time elapsed between administration of pentobarbital sodium and the righting reflex disappearance was recorded as the latency of sleeping time. Time elapsed between disappearance and reappearance of the righting reflex was considered as the duration of sleeping time.

### 4.6. Analysis of Brain Neurotransmitters

Neurotransmitters in the cerebral cortex of the brain, including Glu and GABA, were analyzed by using an UFLC-MS/MS system (Prominence UFLC, Shimadzu, Kyoto, Japan, connected with an 5500 Q-Trap mass detector, Applied Biosystems, Foster city, CA, USA) as previously reported with a slight modification [19,33]. In brief, mice were euthanized after finishing the locomotor activity test, and the cerebral cortex were quickly separated, weighted and conserved at −80 °C until extraction. The thawed cerebral cortex samples were homogenized in 100 μL distilled water on ice, then 50 μL samples were separated and mixed with 100 μL cold acetonitrile, that contained 5 μg/mL 3,4-dihydroxybenzylamine hydrobromide (internal standard). Then the samples were stored in a −80 °C freezer overnight prior to centrifugation at 15,000 *g* for 30 min at 4 °C. 5 μL supernatant was injected to the UFLC-MS/MS system for measurement. Chromatographic separation was performed on a TSKgel Amide 80® column (2 × 150 mm, 3 μm; TOSOH, Tokyo, Japan) maintained at 35 °C. The mobile phase was 60 % water, with 15 mol/L ammonium formate, and acetonitrile at a flow rate of 0.4 mL/min. the mass spectrometer with electrospray ionization source was used in positive ion mode, and the parameters of ionization were as follows: ion source gas 1 and 2, 60 psi; curtain gas, 20 psi; and ion source temperature, 550 °C. The quantification was conducted by multiple reaction monitoring (MRM) of the molecular ion and the related production for each neurotransmitters.

### 4.7. GABA_A_ Receptor Antagonistic Experiment

Two specific GABA_A_ receptor antagonists, bicuculline (2 and 4 mg/kg) and flumazenil (4 and 8 mg/kg), were used to explore whether GABA_A_ receptor participate in the sedative-hypnotic effect of AEO as previous reported [19]. Bicuculline is a light-sensitive competitive antagonist and flumazenil is a benzodiazepine site specific antagonist, which were i.p. administrated 15 min before AEO injection. The locomotor activity test and the hypnotic dose of pentobarbital-induced sleeping test were completed as outlined in Section 4.4 and Section 4.5.

### 4.8. RT-PCR

The mRNA levels of different subunits of GABA_A_ receptor, including α, β, γ and five α variants (α1, α2, α3, α4 and α5) were investigated using RT-PCR. Total RNA extraction, cDNA synthesis and quantitative PCR assays were performed as previously described [34]. Samples were cycled 40 times using a Bio-Rad C1000 Thermal cycler (CFX96 Real-Time System, Bio-Rad, Hercules, CA, USA). Bio-Rad C1000 cycle conditions were as follows: 4 min at 95 °C followed by 40 cycles of 15 s at 95 °C, 20 s at 60 °C and 40 s at 72 °C. Cycle threshold (CT) was calculated under default settings using real-time sequence detection software (Bio-Rad CRX Manager CE 6.0 for Windows, Hercules, CA, USA). At least three independent biological replicates were performed to ensure the reproducibility of the data. The gene-specific primers used for quantitative PCR are listed in Table 2.

### 4.9. Measurement of Cl^−^ Influx in SH-SY5Y Cell

The effects of AEO on intracellular chlorine ion (Cl^−^) level were detected in SH-SY5Y cells as previously reported [13]. SH-SY5Y cells, from the American Type Culture Collection (ATCC, Manassas, VA, USA), were obtained from the Peking Union Medical College (Beijing, China). The cells were cultured in high glucose Dulbecco’s Modified Eagle Medium (DMEM) medium containing 10% fetal bovine serum at 37 °C and 5% carbon dioxide (CO_2_). After reaching 90% confluence, cells were incubated with 5 mM MQAE, a fluorescent indicator for intracellular Cl^−^, which was dissolved in Cl^−^-containing buffer (NaCl 137 mM, KCl 5 mM, NaHCO_3_ 4.2 mM, KH_2_PO_4_ 0.44 mM, MgCl_2_ 1 mM, HEPES 10 mM, glucose 10 mM, CaCl_2_ 1 mM) and was loaded for 45 min at 37 °C. Then the cells were washed with Cl^−^-free buffer before being treated with AEO (0.05, 0.1, 0.2, 0.4 mg/mL) or pentobarbital sodium (0.1 mg/mL) dissolved in Cl^−^-containing buffer for 5 min. After, the buffer was replaced by 2.5 mM nigericin sodium and 3.5 mM tributyltin chloride before the addition of 1.75 mM valinomycin and 10.5 M KSCN to quench the intracellular MQAE fluorescence. Repeated measurement of fluorescence were rapidly recorded by a Tecan Infinite M1000Pro Microplate Reader (TECAN Group Ltd, Shanghai, China) at Ex/Em = 360 nm/450 nm. Intracellular Cl^−^ concentration was calibrated through standard Cl^−^ solutions (0, 10, 20, 30, 40, 50 mM). Stern-Volmer equation F_0_/F_Cl_ = 1 + K_Cl_[Cl^−^] was used to calculate the intracellular Cl^−^ concentration. Where F_0_ represents the maximum fluorescence that can be quenched, F_Cl_ is the increased fluorescence (Ft – Fmin, where Ft is the fluorescence at time 0 or 5 min, and Fmin is the fluorescence after quenching), and K_Cl_ is constant derived from the standard curve.

### 4.10. Statistics Analysis

The results of behavior tests and neurotransmitters measurement were statistically analyzed by two-way analysis of variance (ANOVA) followed by Dunnett’s test using SPSS 17.0 (Chicago, IL, USA), except the result of sub-hypnotic dose pentobarbital-induced sleeping, which was compared using Fisher’s exact test. A one-way ANOVA was performed to the other results. Significance was accepted at *p* < 0.05 and the data are expressed as mean ± SEM.

## 5. Conclusions

In conclusion, this study comprehensively demonstrated that AEO has sedative-hypnotic effects in a dose-dependent manner, which could be related to regulating the GABAergic system. The sedative-hypnotic function of AEO is in part, if not wholly, due to acting on GABA_A_ receptors, promoting the gene transcription, and enhancing Cl^−^ influx. This finding shows that AEO might have promising utility in the prevention and treatment of sleep disorders.

## Figures and Tables

**Table 1:** Chemical compositions and relative amounts of agarwood essential oil (AEO).

No.	Retention Time (min)	Compound	Relative Amount (%)
**1**	7.687	Benzylacetone	1.141
**2**	9.836	5-Acetylpyrimidine	0.173
**3**	11.008	4,6-Di-tert-butyl-*o*-cresol	0.258
**4**	11.453	α-Longifolene	0.308
**5**	11.569	2,3,3,3,4,5-Hexahydro-7-isopropyl-3-methyl-1*H*-cyclopenta[1,3]cyclopropa-[1,2]benzene-3,6 (*7H*)-dione	3.175
**6**	11.788	Selinene	0.326
**7**	11.857	Eremophilene	0.489
**8**	12.008	Guaiene	0.26
**9**	12.065	Guaia-9,11-diene	0.369
**10**	12.233	Verbenol	0.805
**11**	12.36	Espatulenol	0.283
**12**	12.655	Butylated Hydroxytoluene	0.595
**13**	12.92	3-Fluorobenzyl Alcohol	0.331
**14**	13.03	(3*R*)-2,2,5,9-Tetramethyl-3,9-methanodecahydro-1-benzoxepin	0.239
**15**	13.14	Gurjunene	0.112
**16**	13.267	3-Benzylacetylacetone	0.165
**17**	13.446	1-(5,5-Dimethyl-1-cyclopenten-1-yl)-2-methoxybenzene	1.740
**18**	13.856	Diphenyl Disulfide	0.555
**19**	13.972	4-Nitrophthalhydrazide	1.281
**20**	14.249	(–)-Elemene	0.135
**21**	14.451	Sandal	2.996
**22**	14.786	Pyrethrone	2.316
**23**	14.907	Humulene	0.155
**24**	15.075	Farnesene	0.220
**25**	15.306	(+)-Aromadendrene	0.457
**26**	15.439	Spiro[4.4]nonan-2-one	0.321
**27**	15.595	(–)-Cyperene	0.246
**28**	15.849	6-Isopropyl-4,8-α-dimethyl-1,2,3,7,8,8-hexahydronaphthalene	3.481
**29**	16.022	Chamigrene	0.606
**30**	16.259	(3*R*,4*S*,5*R*,8*S*)-5,8-dimethyl-3-(propan-2-yl)-1,2,3,4,4,5,6,8-octahydro-naphthalene	1.891
**31**	16.432	1,1,7-Trimethyl-4-methylenedecahydro-1*H*-cyclopropa[e]azulene	2.874
**32**	16.583	Hinesol	2.745
**33**	16.779	Aristolene	4.063
**34**	17.149	Gualol	14.089
**35**	17.478	(–)-Spathulenol	0.689
**36**	17.767	Germacrene B	3.121
**37**	17.928	2,3,4,5-Tetramethyltricyclo[3.2.1.02,7]oct-3-ene	5.716
**38**	18.067	1-(1,3,4,5,6,7-Hexahydro-4-hydroxy-3,8-dimethylazulen-5-yl)-ethanone	1.046
**39**	18.367	Gurjunene	0.707
**40**	18.5	Chiloscyphone	1.486
**41**	18.754	2-Cyclohexylanisole	6.208
**42**	18.893	Eudesmene	1.074
**43**	19.124	Santolina Triene	1.450
**44**	19.401	4,11,11-Trimethyl-8-methylene-bicyclo[7.2.0]-undec-4-ene	0.201
**45**	19.621	Longifolenaldehyde	0.544
**46**	19.765	Ledol	0.388
**47**	19.904	3-Tetradecyne	0.213
**48**	20.158	3-Ethylpyridine oxide	1.310
**49**	20.337	1-Cyclohexeneethanol	0.553
**50**	20.805	4-Methoxycoumarin	0.737
**51**	20.949	Alloaromadendrene	0.140
**52**	21.261	Perhydropyrene	1.619
**53**	21.371	*o*-*tert*-Butylphenol	1.100
**54**	21.654	Isodurol	0.880
**55**	22.105	Aristol-9-en-8-one	0.849
**56**	22.405	Valencene	0.686
**57**	22.752	Xanthotricin	0.225
**58**	23.116	2,4,6-Triisopropylbenzoic Acid	0.265
**59**	24.086	Dehydrofukinone	4.096
**60**	24.721	1-Heptatriacotanol	0.111
**61**	25.698	5-(2-Thienyl)-4-pyrimidinamine	0.414
**62**	28.418	2-Ethylphenol	0.166
**63**	30.226	2-Propylthiophene	0.124
**64**	30.787	Methyleugenol	0.408
**65**	31.832	Dispiro[5.1.5.3]hexadecan-7-one	0.205
**66**	32.173	Dibutyl phthalate	0.291
**67**	33.536	7-Methyltridecane	11.228
**68**	34.836	Trans-*N*,*N*′-diferuloylputrescine	0.794
Total:			98.244

**Table 2:** Primers used in quantitative real-time polymerase chain reaction (RT-PCR) analysis.

Name	Forward (5′-3′)	Reverse (3′-5′)
α	TGGACTCCTGATACNTTYTT	GCHATRAACCARTCCATGGC
β	CTGGATGARCAAAACTGYAC	ACAAAGACAAARCAWCCCAT
γ	TAGACAGCAAYATGGTGGG	TTGATCCAAAADGACACCCAGG
α_1_	AAAAGTCGGGGTCTCTCTGAC	CAGTCGGTCCAAAATTCTTGTGA
α_2_	GGACCCAGTCAGGTTGGTG	TCCTGGTCTAAGCCGATTATCAT
α_3_	ATGTGGCACTTTTATGTGACCA	CCCCAGGTTCTTGTCGTCTTG
α_4_	ACAATGAGACTCACCATAAGTGC	GGCCTTTGGTCCAGGTGTAG
α_5_	TGACCCAAACCCTCCTTGTCT	GTGATGTTGTCATTGGTCTCGT
β-actin	GGCTGTATTCCCCTCCATCG	CCAGTTGGTAACAATGCCATGT
